# Identifying risk factors for vasculogenic etiology in patients with erectile dysfunction based on clinical features and machine learning

**DOI:** 10.3389/fendo.2026.1849563

**Published:** 2026-05-21

**Authors:** Jian Wang, Yancheng Wu, Xiaoyan Zhang, Yang Lu, Zhenrong Piao, Wei Zhao, Maosen Zhang

**Affiliations:** 1 Department of Andrology, Affiliated Hospital of Nanjing University of Chinese Medicine, Nanjing, China; 2 Department of Andrology, The First Clinical Medical College of Nanjing University of Chinese Medicine, Nanjing, China; 3 Department of Andrology, Huai’an Hospital of Traditional Chinese Medicine, Huai’an, China; 4 School of Medicine, Nanjing University of Chinese Medicine, Nanjing, China; 5 Department of Urology, Yangzhou Hospital of Traditional Chinese Medicine, Yangzhou, China

**Keywords:** color Doppler duplex ultrasonography, machine learning, risk factors, Shapley Additive exPlanations, vasculogenic erectile dysfunction

## Abstract

**Background:**

Vasculogenic erectile dysfunction (ED) is an important subtype of organic ED, and its development and progression are closely related to endocrine, metabolic, and psychological factors. Identifying risk factors for vasculogenic ED may facilitate early recognition and targeted intervention.

**Methods:**

This study included 519 patients diagnosed with ED using penile color Doppler duplex ultrasonography (CDDU) as the gold standard. Clinical and laboratory indicators were collected. Feature selection was strictly performed within the training set using univariate logistic regression, the Boruta algorithm, and least absolute shrinkage and selection operator (LASSO) regression. Based on the selected key variables, five machine learning models—logistic regression, random forest, support vector machine, light gradient boosting machine (LightGBM), and extreme gradient boosting (XGBoost)—were constructed and compared. Model performance was evaluated using metrics including the area under the receiver operating characteristic curve (AUC), and the SHapley Additive exPlanations (SHAP) method was employed to interpret the optimal model.

**Results:**

Among the 519 patients, 235 were diagnosed with vasculogenic ED. Feature selection identified seven key risk factors: age, hypertension, smoking, diabetes, Hamilton Anxiety Scale (HAMA) score, total testosterone (T), and estradiol (E2). The random forest model performed best in the validation set, but its discriminative ability was only moderate (AUC = 0.682, 95% confidence interval [CI]: 0.598–0.768). SHAP analysis revealed that age contributed most to the model predictions, followed by hypertension, T, and smoking; the HAMA score also ranked highly. Testosterone levels exhibited a nonlinear U−shaped association with vasculogenic ED risk.

**Conclusion:**

Based on routine clinical indicators, this study identified seven key factors associated with vasculogenic ED. Among them, anxiety as measured by the HAMA score was recognized as a non−traditional factor, suggesting a complex interplay between psychological factors and vascular pathology; however, the specific direction of this relationship remains to be elucidated by prospective studies. The machine learning model constructed in this study showed moderate discriminative ability and is currently insufficient to support independent clinical decision−making. Future research should collect multicenter, large−sample data and adjust model parameters for further validation and optimization.

## 1 Introduction

Erectile dysfunction (ED) is a common condition that severely impairs men’s quality of life. Its prevalence has risen continuously in recent years, making it an important public health issue (1). Among the organic forms of ED, vasculogenic etiology is the most common, with the core pathological mechanisms being insufficient penile arterial perfusion and/or abnormal veno-occlusive function (2). Currently, the gold standard for diagnosing vasculogenic ED is penile color Doppler duplex ultrasonography (CDDU) performed after intracavernous injection of vasoactive agents. However, this examination is invasive, demands a high level of technical expertise, and may induce anxiety and discomfort in patients, thereby limiting its widespread use in routine clinical evaluation (3). Therefore, exploring modifiable risk factors closely associated with the disease and identifying high-risk populations for vasculogenic ED are of great significance for optimizing the diagnostic and therapeutic pathway and for rationally selecting candidates for CDDU.

Previous studies have confirmed that metabolic and psychological factors such as hypogonadism, obesity, dyslipidemia, anxiety, and depression are significantly associated with the onset and aggravation of ED, and complex interactions exist among these factors (2–4). Traditional statistical methods, such as univariate analysis or multivariate logistic regression, have limited ability to capture such nonlinear and multifactorial synergistic effects, and are insufficient to fully reveal the combined risk effects of multidimensional clinical indicators on vasculogenic ED.

In recent years, machine learning techniques, with their advantages in handling high-dimensional and nonlinear data, have shown great promise in the field of disease risk factor identification (5, 6). These methods can automatically learn complex mapping relationships among variables, providing a new technical approach for screening key risk factors from multidimensional clinical data. Moreover, model interpretation tools such as Shapley Additive exPlanations (SHAP) can quantify the contribution of each feature to individual risk, thereby helping to enhance model credibility and explore its biological plausibility (7, 8). On this basis, the present study aimed to preliminarily evaluate common clinical factors associated with vasculogenic ED. By constructing and comparing several mainstream machine learning algorithms, we sought to explore data-driven association patterns and provide preliminary evidence for the identification and precise screening of high-risk populations for vasculogenic ED.

## 2 Materials and methods

### 2.1 Study design and participants

This study was a single-center, retrospective, cross-sectional observational study. To ensure sample continuity and reduce selection bias, we retrospectively included all 519 eligible patients with ED who were hospitalized in the Andrology Department of Jiangsu Province Hospital of Chinese Medicine from June 1, 2024, to December 31, 2024, and completed penile CDDU and related biochemical tests. The specific enrollment process was as follows: all consecutive patients who met the following inclusion and exclusion criteria and completed the CDDU examination during the study period were enrolled without any additional selection criteria. The study protocol was approved by the Ethics Committee of Jiangsu Province Hospital of Chinese Medicine (approval number: 2024NL-309-01), and informed consent was obtained from all participants. Inclusion criteria were male patients aged 18–69 years presenting with a chief complaint of ED. Exclusion criteria included ED caused by definite neurological disorders, a history of pelvic surgery or trauma, severe mental illness, current use of antiandrogen medications, or recent hormone therapy.

### 2.2 Data collection and outcome definition

All patients underwent standardized assessments, including medical history taking, physical examination, laboratory tests, and psychological scale evaluations. Fasting venous blood samples were collected in the morning after admission, and the following indicators were measured: insulin-like growth factor-1 (IGF-1), uric acid (UA), low-density lipoprotein cholesterol (LDL), high-density lipoprotein cholesterol (HDL), triglycerides (TG), total cholesterol (TC), total testosterone (T), estradiol (E2), luteinizing hormone (LH), follicle-stimulating hormone (FSH), prolactin (PRL), alanine aminotransferase (ALT), and aspartate aminotransferase (AST). Body mass index (BMI) was calculated from measured height and weight. Lifestyle variables included smoking (Smoke, 1 = yes/0 = no) and alcohol consumption (Drunk, 1 = yes/0 = no). Chronic disease history included hypertension (Hypertension, 1 = yes/0 = no) and diabetes mellitus (DM, 1 = yes/0 = no). Psychological status was assessed using the Hamilton Depression Scale (HAMD) and the Hamilton Anxiety Scale (HAMA). CDDU-related parameters were collected strictly according to standard operating procedures, including bilateral cavernosal artery peak systolic velocity (PSV), end-diastolic velocity (EDV), and resistance index (RI). The diagnosis of vasculogenic ED was based on ultrasound criteria from previous studies: unilateral cavernous artery PSV < 25 cm/s or a bilateral sum of PSV < 50 cm/s indicated arterial insufficiency; additionally, an EDV > 5 cm/s suggested veno-occlusive dysfunction. Patients who met either of the above criteria were diagnosed with vasculogenic ED (9, 10). According to these criteria, patients were divided into a vasculogenic ED group and a non-vasculogenic ED group.

### 2.3 Data preprocessing and feature selection

The entire dataset was randomly split into a training set (n = 363) and an independent validation set (n = 156) at a ratio of 7:3. A total of 21 initial variables were included in this study. After verification, no missing data were found; therefore, no imputation was performed. All feature selection steps were strictly conducted within the training set. Three complementary strategies were employed for feature selection (11): (a) univariate logistic regression analysis (with a significance threshold of P<0.05); (b) the Boruta algorithm, based on random forest, to identify all relevant features; and (c) LASSO regression, in which the optimal regularization parameter λ was determined through 10-fold cross-validation, and features with non-zero coefficients were selected. The intersection of the features retained by the three methods on the training set was taken to determine the final variables entering the machine learning models.

### 2.4 Construction and training of machine learning models

On the aforementioned training set, five machine learning classifiers were constructed and compared: 1) Logistic regression, serving as the baseline reference; 2) Random forest, an ensemble model based on decision trees, used to handle nonlinear relationships; 3) Support vector machine, with a radial basis function kernel; 4) LightGBM; and 5) XGBoost. All models were trained with default parameters, without additional hyperparameter optimization or class imbalance handling, to ensure baseline consistency in model comparison.

### 2.5 Model evaluation and interpretability

The predictive performance of each model was evaluated on the independent validation set. The primary evaluation metric was the area under the receiver operating characteristic curve (AUC) and its 95% confidence interval (CI), which was calculated using the bootstrap method with 2000 resamples. Secondary metrics included accuracy, sensitivity, specificity, precision, F1 score, and G-mean. The random forest model, which demonstrated the best overall performance on the validation set, was selected for SHAP interpretation. SHAP values were used to quantify the contribution of each feature to individual predictions. A SHAP summary plot was employed to display the global importance of features, and SHAP dependence plots were used to reveal the association patterns between key features and predicted risk.

### 2.6 Statistical analysis

The normality of continuous variables was assessed using the Shapiro–Wilk test. For baseline characteristics: normally distributed continuous variables were expressed as mean ± standard deviation and compared using the independent samples *t*-test; non-normally distributed continuous variables were expressed as median (interquartile range) and compared using the Mann–Whitney U test; categorical variables were expressed as frequencies (percentages) and compared using the chi-square test or Fisher’s exact test. All statistical analyses and machine learning modeling were performed using SPSS 26.0 and R software (version 4.5.1), respectively.

## 3 Results

### 3.1 Baseline characteristics of the study cohort

**Figure 1:**
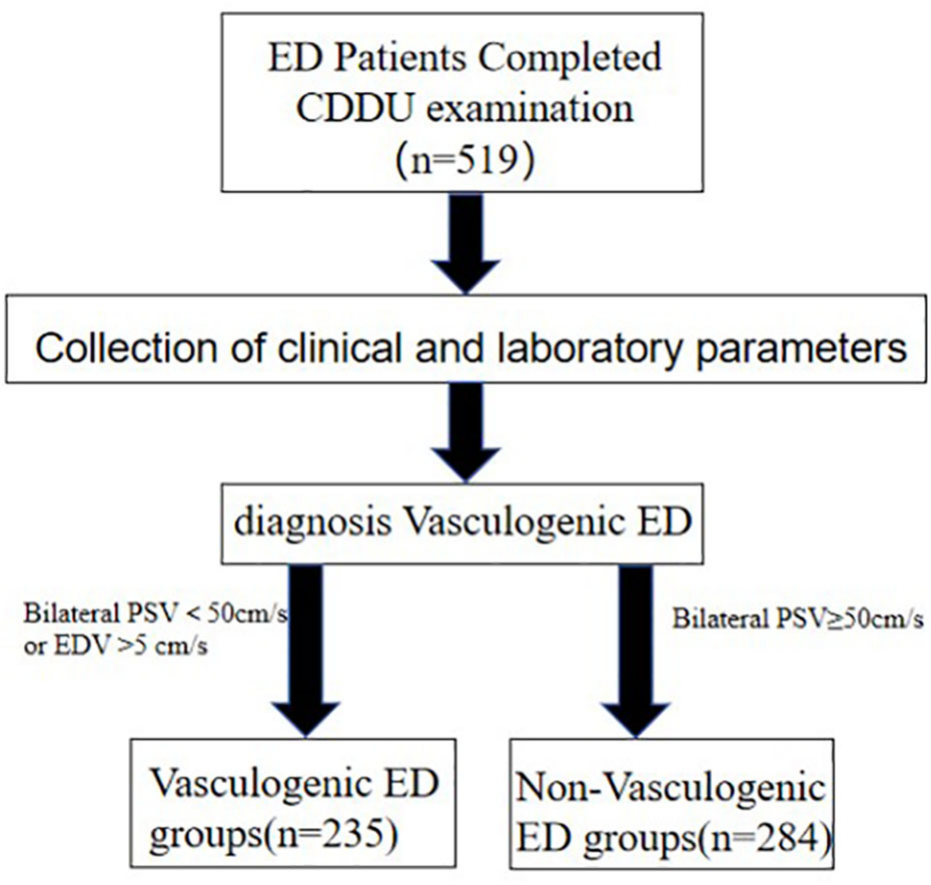
Study flowchart.

A total of 519 adult patients with ED were included in this study. Among them, 235 were diagnosed with vasculogenic ED by penile CDDU, and 284 were classified as non-vasculogenic ED. The flowchart is shown in **Figure 1**. All continuous variables were non-normally distributed (Shapiro–Wilk test, *P* < 0.05); therefore, the Mann–Whitney U test was used for comparisons. The baseline clinical characteristics of the two groups are presented in **Table 1**. Compared with the non-vasculogenic ED group, patients in the vasculogenic ED group were older, had higher FSH levels, and exhibited a higher prevalence of smoking history, hypertension, and diabetes mellitus, whereas HAMA scores, serum total T, and E2 levels were significantly lower. The two groups did not differ significantly in BMI, the four lipid parameters (total cholesterol, triglycerides, low-density lipoprotein cholesterol, and high-density lipoprotein cholesterol), LH, PRL, HAMD score, or alcohol consumption history (all *P* > 0.05).

**Table 1:** Baseline characteristics of the study participants.

Variables	Total (n=519)	Without vasculogenic ED (n=284)	With vasculogenic ED (n=235)	P value
Bilateral PSV (cm/s)	53.70 (35.10, 69.80)	68.70 (60.13, 78.70)	33.42 (32.46, 34.37)	<0.001
Left PSV (cm/s)	26.00 (16.60, 35.50)	34.45 (28.13, 41.10)	15.80 (13.10, 19.10)	<0.001
Left EDV (cm/s)	0.40 (0.36, 1.62)	0.39 (0.35, 0.43)	1.10 (0.38, 3.26)	<0.001
Left RI	0.98 (0.92, 0.99)	0.98 (0.98, 0.99)	0.93 (0.78, 0.97)	0.006
Right PSV (cm/s)	25.90 (17.00, 34.50)	33.65 (28.63, 40.75)	16.30 (13.40, 19.40)	<0.001
Right EDV (cm/s)	0.43 (0.38, 2.78)	0.42 (0.38, 0.51)	2.02 (0.40, 3.77)	<0.001
Right RI	0.97 (0.87, 0.98)	0.98 (0.98, 0.99)	0.87 (0.78, 0.97)	<0.001
Age (years)	35.00 (30.00, 43.00)	32.00 (28.00, 38.00)	38.00 (32.00, 48.00)	<0.001
ALT (U/L)	22.00 (14.00, 32.00)	23.00 (15.00, 34.00)	21.00 (14.00, 30.00)	0.093
AST (U/L)	20.00 (17.00, 25.00)	20.00 (18.00, 25.00)	20.00 (17.00, 25.00)	0.320
IGF-1(ng/mL)	168.00 (135.00, 201.00)	171 (138.25, 203.00)	161.00 (133.00, 195.00)	0.029
BMI (kg/m²)	25.06 (22.76, 27.68)	24.80 (22.41, 27.46)	25.48 ± 3.19	0.100
UA(μmol/L)	371.00 (318.00, 419.00)	377.50 (324.25, 420.75)	369.00 (311.00, 417.00)	0.112
LDL (mmol/L)	2.86 (2.42, 3.33)	2.91 ± 0.74	2.86 (2.35, 3.32)	0.600
HDL (mmol/L)	1.07 (0.91, 1.25)	1.08 (0.92, 1.26)	1.05 (0.90, 1.24)	0.505
TG (mmol/L)	1.42 (0.94, 2.09)	1.43 (0.91, 2.04)	1.40 (0.95, 2.11)	0.447
TC (mmol/L)	4.41 (3.89, 4.97)	4.43 ± 0.86	4.44 (3.91, 5.06)	0.373
T (ng/dL)	432.00 (425.44, 455.84)	452.58 (354.96, 563.24)	403.20 (285.92, 552.96)	0.001
E2 (ng/dL)	30.00 (24.00, 37.00)	31.00 (25.00, 37.75)	29.00 (22.00, 35.00)	0.012
LH (mIU/mL)	5.50 (5.19, 7.19)	5.42 (4.16, 6.80)	5.69 (4.30, 7.67)	0.077
FSH (mIU/mL)	4.37 (3.13, 6.02)	4.19 (3.00, 5.68)	4.60 (3.31, 6.84)	0.003
PRL (ng/dL)	18.71 (14.38, 23.97)	18.71 (14.92, 23.52)	18.57 (13.72, 25.15)	0.691
HAMD (score)	11.00 (6.00, 17.00)	12.00 (7.00, 17.75)	10.00 (6.00, 17.00)	0.171
HAMA (score)	12.00 (8.00, 18.00)	12.00 (9.00, 19.00)	11.00 (7.00, 16.00)	0.001
Smoke				<0.001
0	396 (76.3%)	244 (85.9%)	152 (64.7%)	
1	123 (23.7%)	40 (14.1%)	83 (35.3%)	
Drunk				0.234
0	354 (68.2%)	200 (70.4%)	154 (65.5%)	
1	165 (31.8%)	84 (29.6%)	81 (34.5%)	
Hypertension				<0.001
0	419 (80.7%)	257 (90.5%)	162 (68.9%)	
1	100 (19.3%)	27 (9.5%)	73 (30.1%)	
DM				<0.001
0	471 (90.8%)	271 (95.4%)	200 (85.1%)	
1	102 (9.2%)	13 (4.6%)	35 (14.9%)	

Bilateral PSV, bilateral peak systolic velocity; Left PSV, left peak systolic velocity; Left EDV, left end-diastolic velocity; Left RI, left resistance index; Right PSV, right peak systolic velocity; Right EDV, right end-diastolic velocity; Right RI, right resistance index; Age, age; ALT, alanine aminotransferase; AST, aspartate aminotransferase; IGF-1, insulin-like growth factor-1; BMI, body mass index; UA, uric acid; LDL, low-density lipoprotein; HDL, high-density lipoprotein; TG, triglycerides; TC, total cholesterol; T, testosterone; E2, estradiol; LH, luteinizing hormone; FSH, follicle-stimulating hormone; PRL, prolactin; HAMD, Hamilton Depression Rating Scale; HAMA, Hamilton Anxiety Rating Scale; Smoke, smoking history; Drunk, drinking history; Hypertension, history of hypertension; DM, history of diabetes mellitus.

### 3.2 Feature selection results

**Figure 2:**
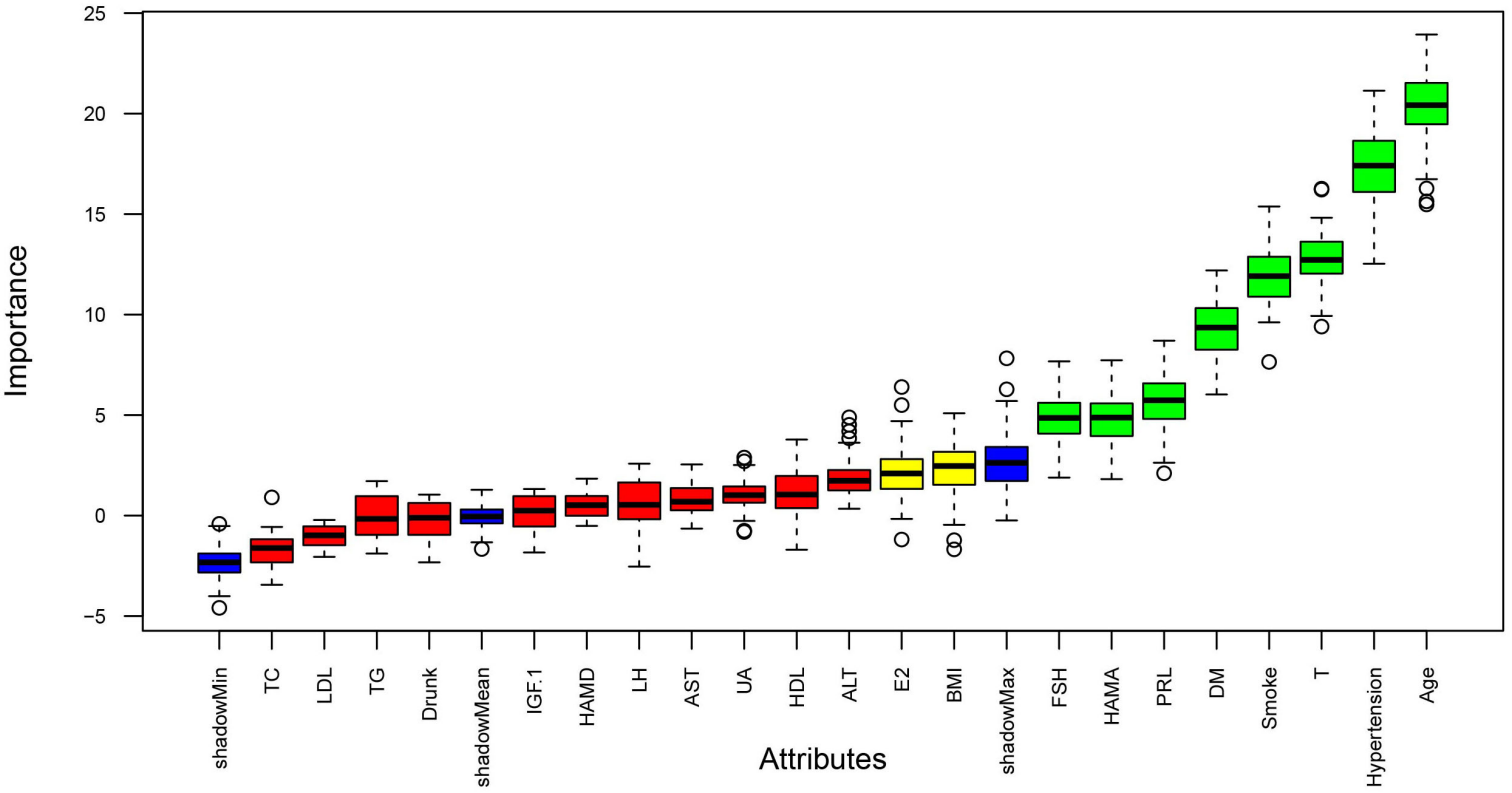
Boruta feature importance plot. The output of the Boruta algorithm is shown in the figure, where the blue lines represent the shadow scores (minimum, mean, and maximum). Variables are classified according to the color of the boxplots: green for important variables, yellow for tentative variables, and red for rejected variables.

**Figure 3:**
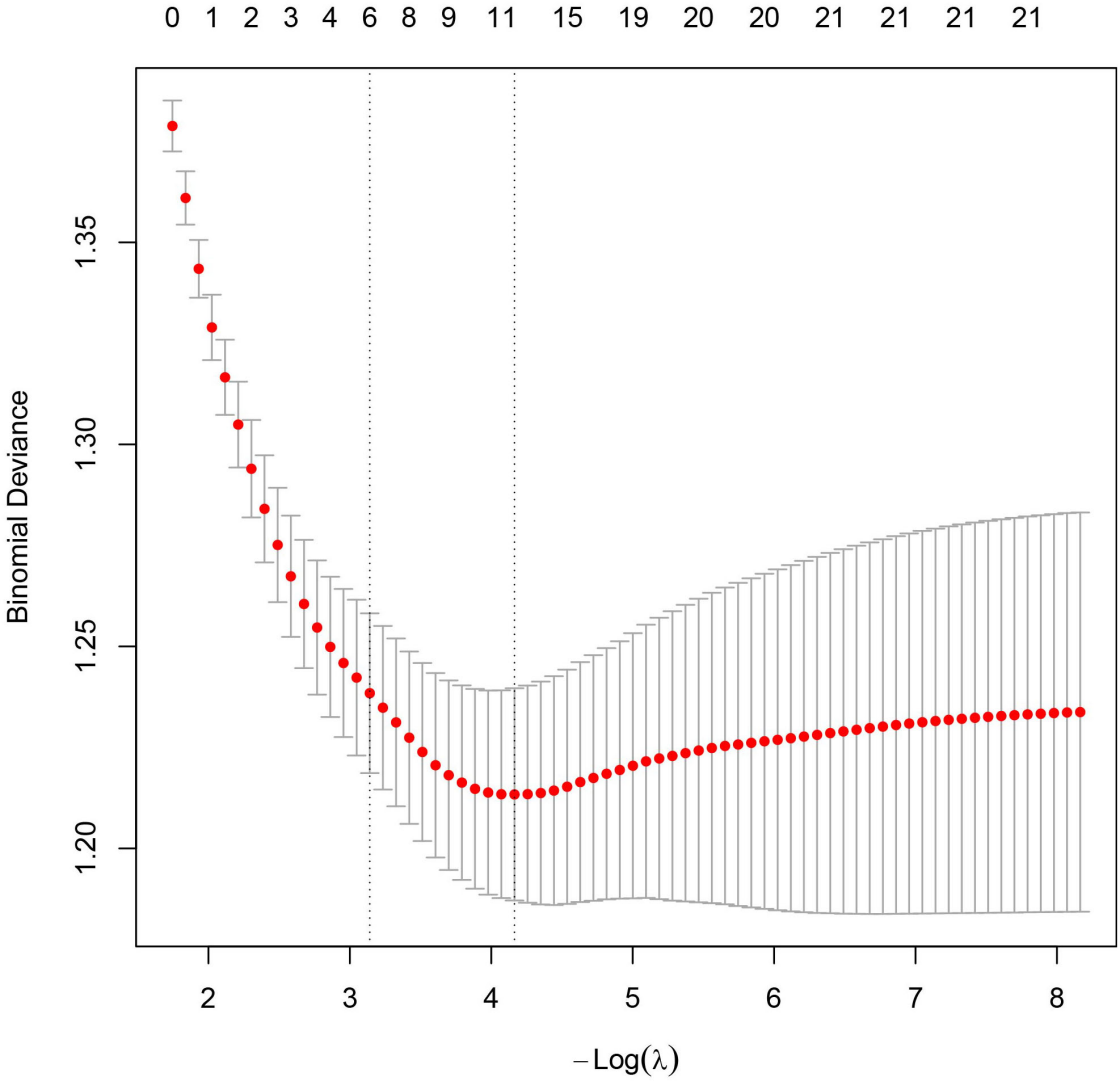
LASSO cross - validation error plot. The relationship between the λ value and binomial deviance is shown in the figure. Two vertical dashed lines in the figure mark two key positions: the left dashed line corresponds to the minimum mean squared error, and the right dashed line corresponds to the position of the minimum mean squared error plus one standard error.

**Figure 4:**
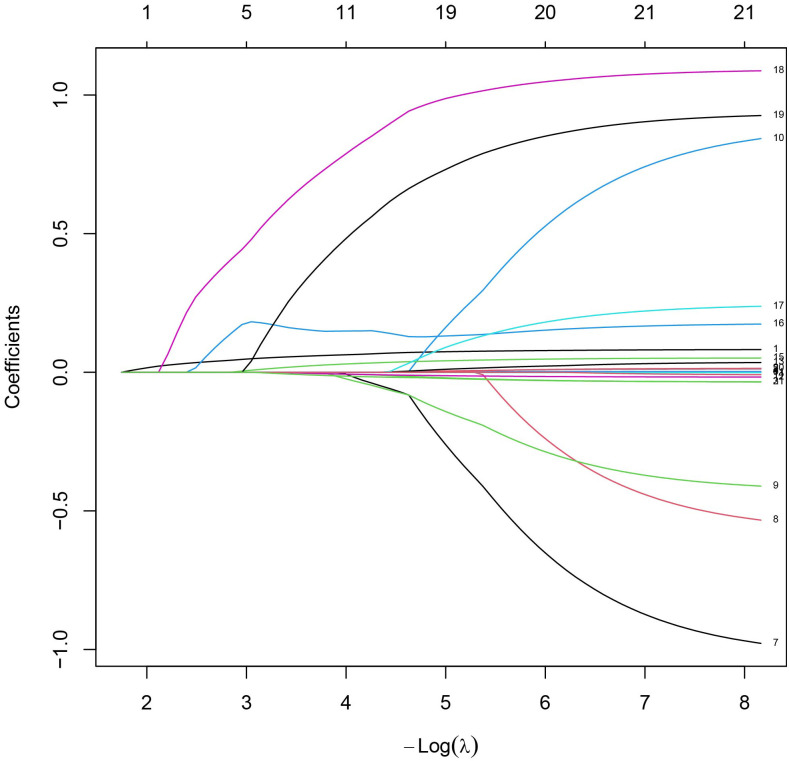
LASSO coefficient path plot. The figure illustrates the relationship between the L1 norm (i.e., the regularization term of the model) and the individual regression coefficients in the LASSO regression.

Univariate analysis identified Age, Hypertension, Smoke, DM, FSH, HAMA, T, E2, IGF−1, and LH (P < 0.05). The Boruta algorithm identified 11 features associated with vasculogenic ED: Age, ALT, BMI, T, E2, FSH, PRL, Smoke, Hypertension, DM, and HAMA (**Figure 2**). LASSO regression with cross−validation selected Age, AST, IGF−1, LDL, TG, T, E2, PRL, Smoke, Hypertension, DM, and HAMA (**Figure 3**, **4**). The intersection of the three methods finally determined seven key predictors for inclusion in the models: Age, Hypertension, Smoke, DM, HAMA, T, and E2. The remaining variables were not included in the final models.

### 3.3 Performance comparison of machine learning models

**Figure 5:**
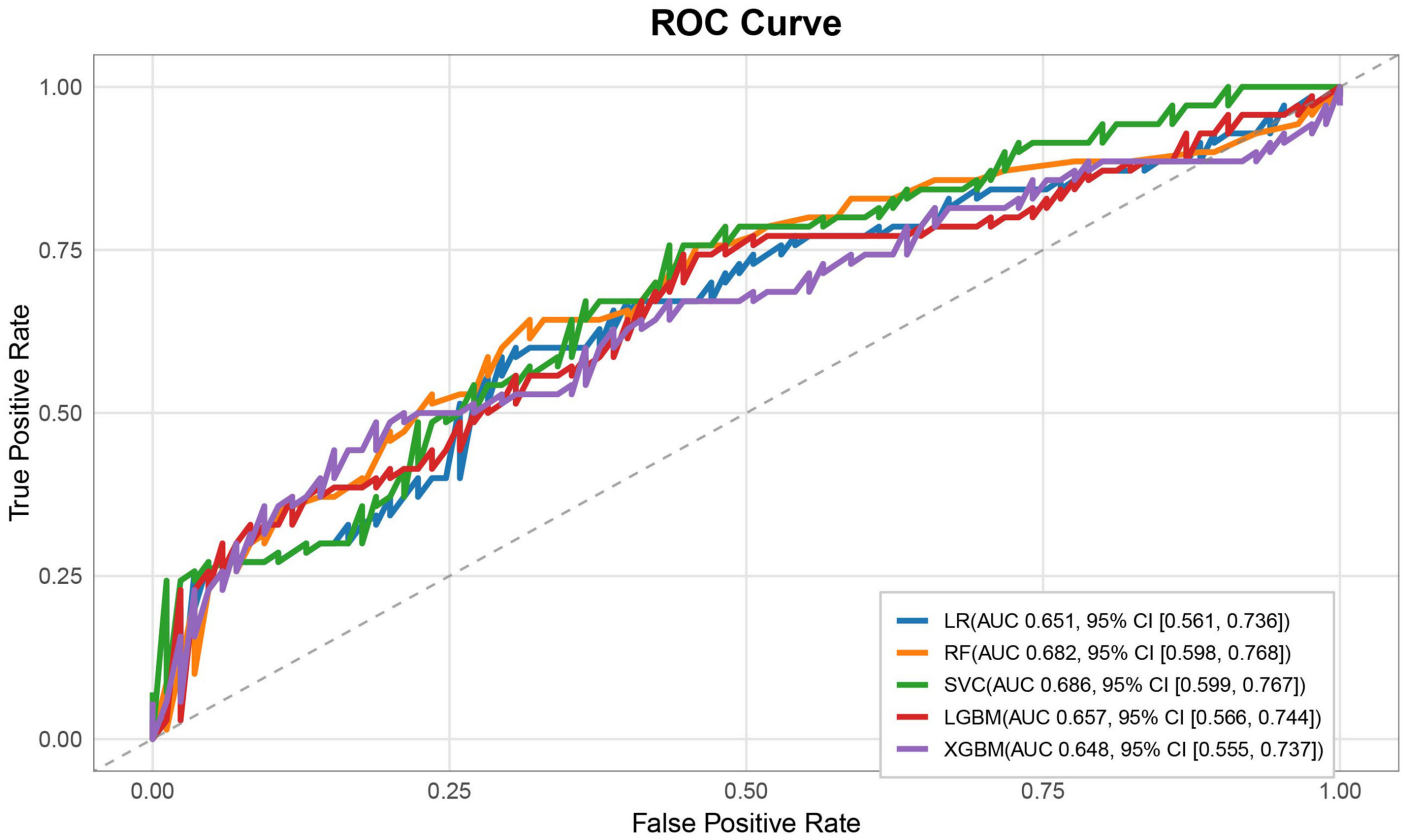
ROC curves of multiple models. The ROC analysis results of various machine learning models for vascular ED are shown in the figure. Among them, AUC is the area under the curve; LR is logistic regression; RF is random forest; SVC is support vector classification; LGBM is light gradient boosting machine; XGBM is extreme gradient boosting machine.

On the independent validation set (n = 156), the predictive performance of each model is shown in **Table 2**. **Figure 5**. The random forest (RF) model achieved relatively balanced performance (AUC = 0.682, 95% CI: 0.598–0.768), with stable accuracy (0.639), sensitivity (0.528), specificity (0.729), and F1 score (0.569). Although the support vector classifier (SVC) had a slightly higher AUC (0.686), its specificity (0.271) and accuracy (0.368) were substantially lower, suggesting overfitting or an unstable decision boundary. Considering all metrics comprehensively, the RF model was selected for subsequent interpretability analysis.

**Table 2:** Performance metrics of each model on the validation set.

Model	AUC	Accuracy	Sensitivity	Specificity	Precision	F1_Score	G_mean
LR	0.651	0.613	0.457	0.741	0.593	0.516	0.582
RF	0.682	0.639	0.528	0.729	0.617	0.569	0.621
SVC	0.686	0.368	0.486	0.271	0.354	0.410	0.363
LGBM	0.657	0.613	0.586	0.635	0.569	0.577	0.610
XGBM	0.648	0.626	0.529	0.706	0.597	0.561	0.611

LR, Logistic regression; RF, Random forest; SVC, Support vector classification; LGBM, Light gradient boosting machine; ML, machine learning; XGBM, eXtreme gradient boosting machine.

### 3.4 Model interpretability: SHAP analysis

**Figure 6:**
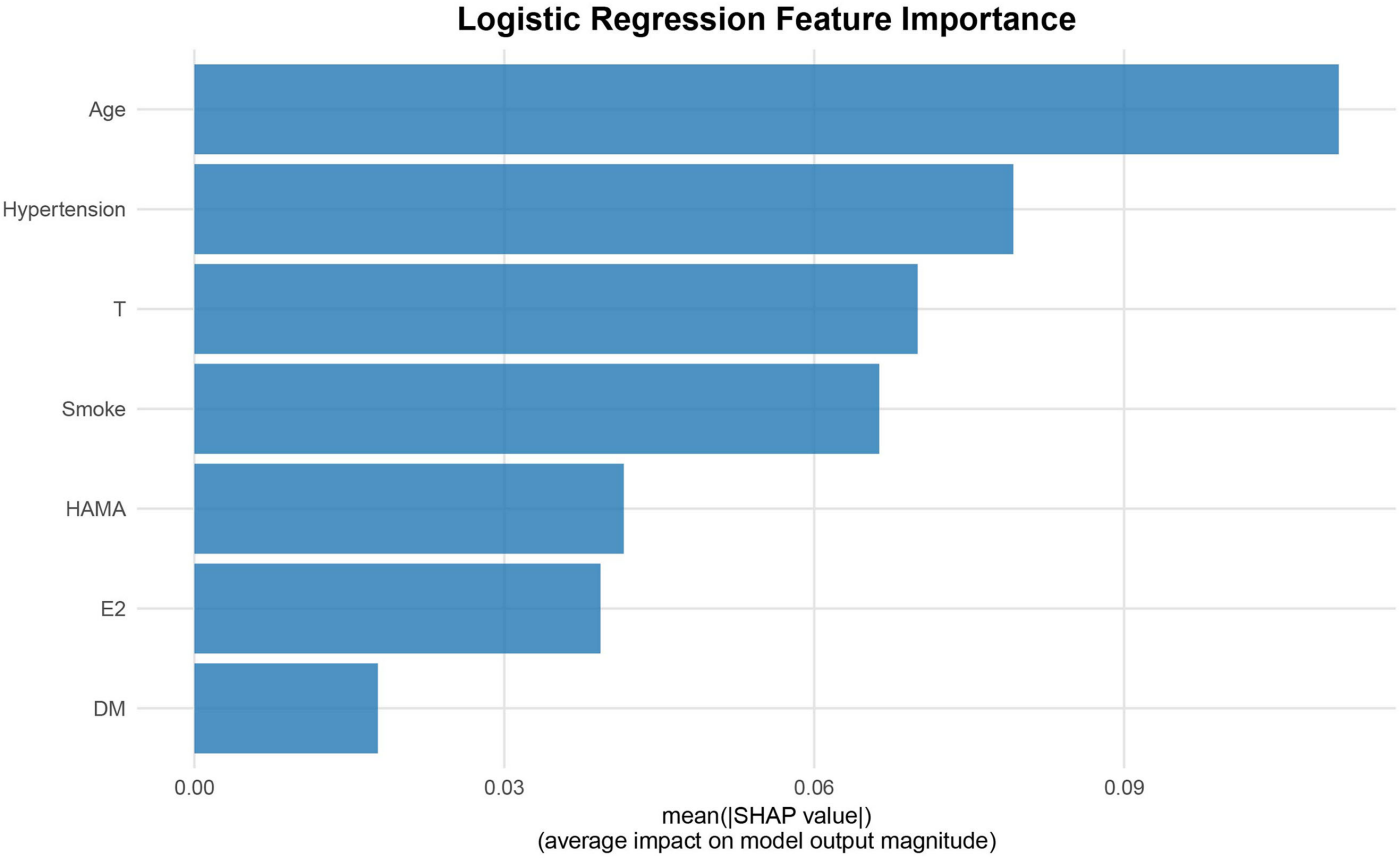
SHAP feature importance bar plot. The figure displays the SHAP importance of seven features in the logistic regression model in descending order, measured by the mean absolute SHAP value of each feature to reflect its impact on the model’s prediction. A longer bar indicates a greater contribution and higher importance of the variable.

**Figure 7:**
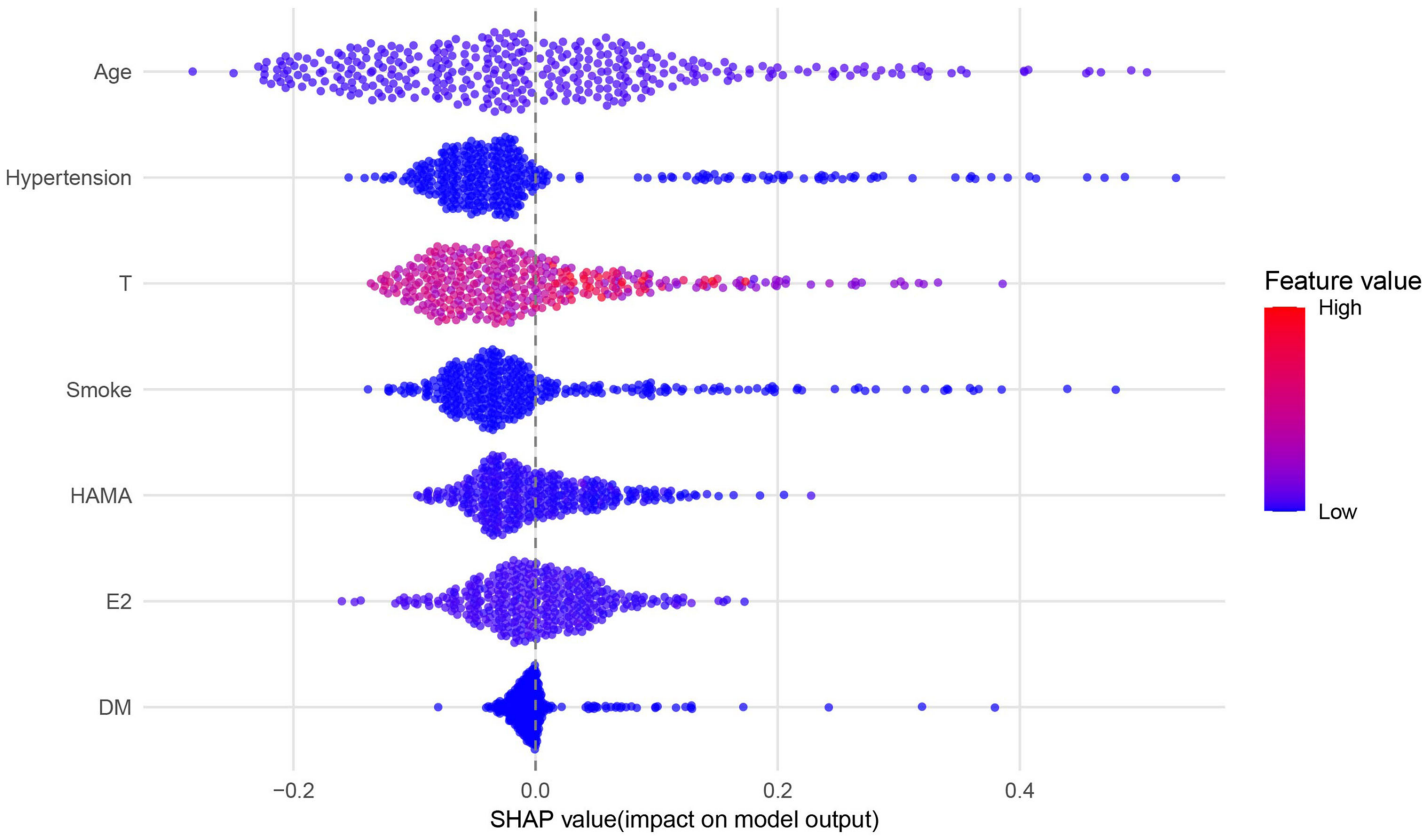
SHAP beeswarm plot. Each dot represents an individual sample. The horizontal axis represents the SHAP value, reflecting the feature’s impact on the model output: positive values increase the predicted risk, while negative values decrease it. The color gradient (from blue to red) corresponds to the feature value, ranging from low to high. Features are ordered by the magnitude of their average SHAP value, reflecting their overall importance to the model.

**Figure 8:**
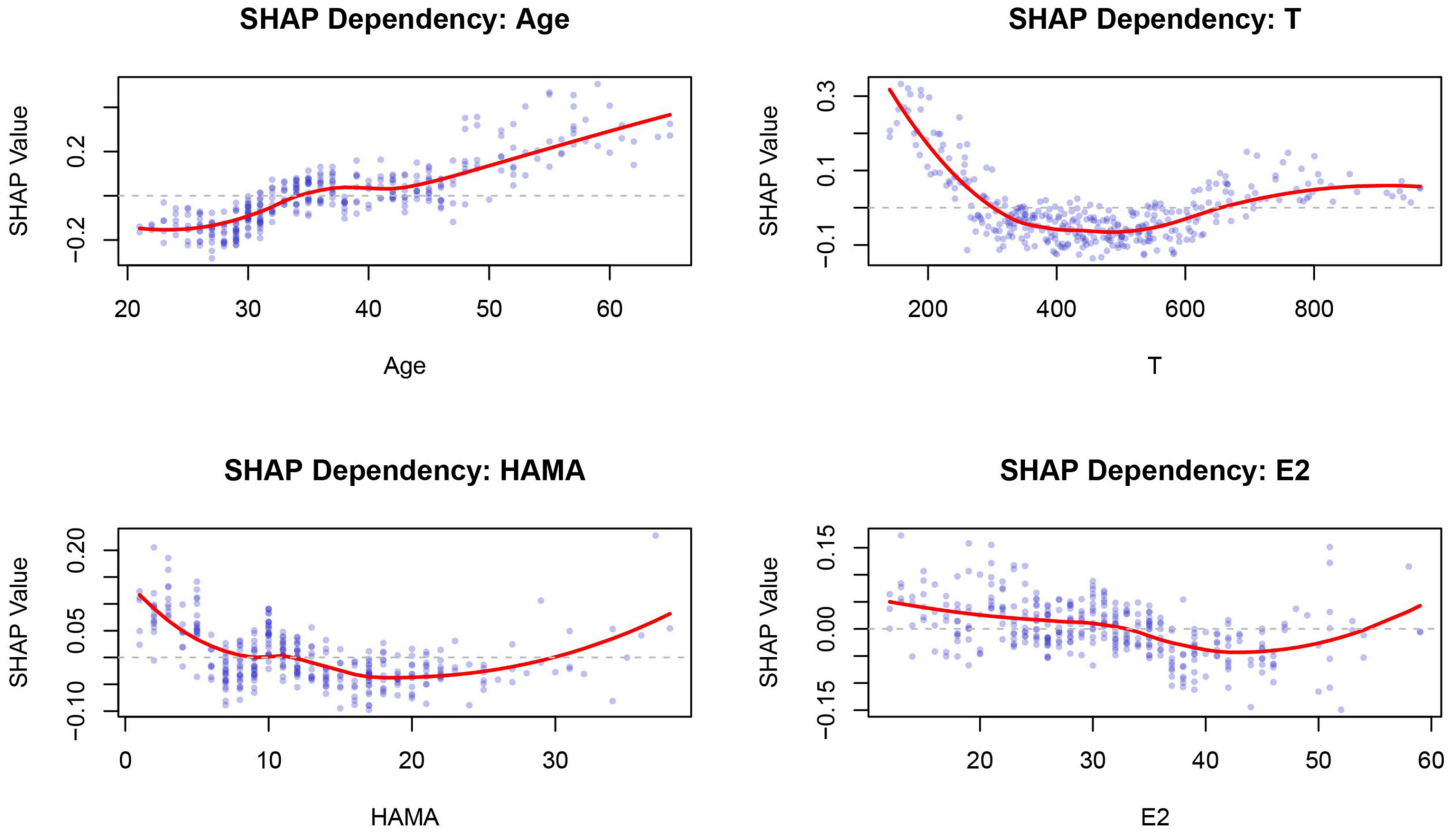
SHAP dependence plot combination. The x-axis represents feature values, and the y-axis represents SHAP values (impact on model output). Blue points represent individual samples, and the red line indicates the trend. Positive values increase the predicted risk, while negative values decrease it.

**Figure 9:**
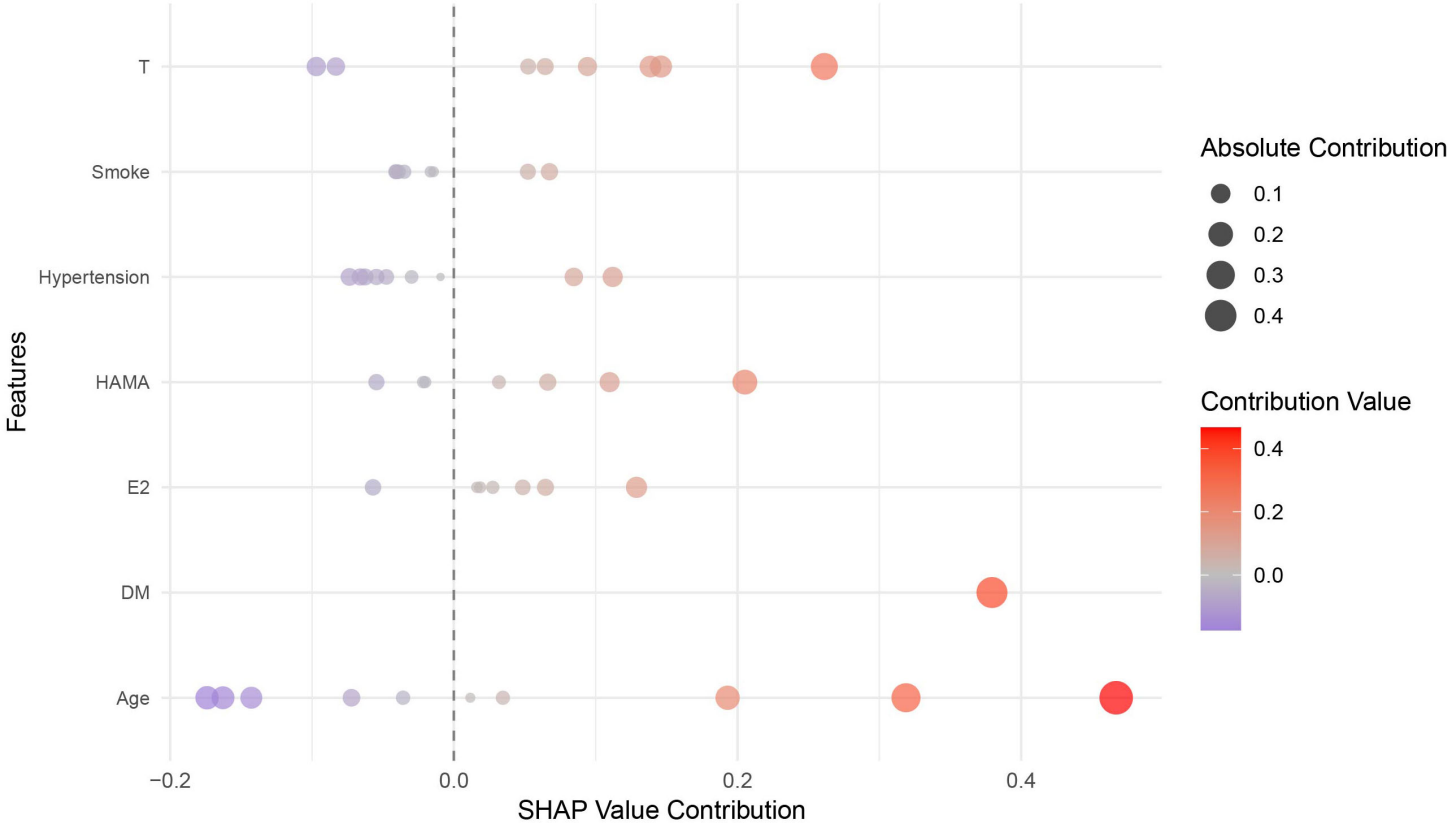
Scatter plot of SHAP contributions for multiple samples. Each point represents an individual sample. The horizontal axis is the SHAP value, where positive values increase the predicted risk and negative values decrease it. The color of each point indicates the direction of the contribution value, and its size reflects the magnitude of the absolute contribution. Features are listed in descending order of their impact on the model.

The SHAP analysis revealed that age was the feature contributing most to the model output, with the widest distribution of SHAP values, indicating that this feature was most closely associated with individual predicted risk. The remaining features, in descending order of importance, were hypertension, T, smoking, HAMA score, E2, and DM (**Figure 6**, **7**). SHAP dependence plots further suggested nonlinear association patterns between the predicted risk and total testosterone, HAMA score, and estradiol (**Figure 8**). Specifically, the T level exhibited a U-shaped association with vasculogenic ED risk: both low and excessively high levels were associated with elevated risk, whereas a moderate level showed a relative protective trend. Age displayed a positive contribution with relatively low inter-individual variability. DM also exerted a notable positive driving effect. The feature points for smoking and hypertension were mostly distributed to the right of the zero line of the SHAP values, suggesting that both tended to be associated with increased risk, although the inter-individual heterogeneity was appreciable (**Figure 9**). The contribution of E2 showed a bidirectional distribution pattern.

## 4 Discussion

Based on single-center retrospective cross-sectional data, this study integrated multiple machine learning algorithms to construct a prediction model for vasculogenic ED aimed at exploring associated factors. The results showed that the RF model performed relatively better than logistic regression and some ensemble models on the validation set (AUC = 0.682), but its predictive ability remained moderate. Owing to its ensemble of decision trees and bootstrap aggregating mechanism, RF exhibits good robustness to nonlinear interactions among features and to outliers, and was therefore selected for interpretation. Through SHAP analysis, we identified seven features strongly associated with vasculogenic ED: age, hypertension, smoking, diabetes mellitus, total testosterone, estradiol, and HAMA score.

In the ranking of feature importance, age was the strongest factor driving model predictions, with the widest distribution of SHAP values and low inter-individual variability, a finding highly consistent with existing epidemiological evidence (12). From a pathophysiological perspective, the major factors in age-related ED include reduced nitric oxide (NO) bioavailability due to endothelial oxidative stress and inflammation, decreased cavernous smooth muscle content, and increased arterial stiffness caused by metabolic diseases (13–16). Chronic inflammation, oxidative stress, hormonal imbalance, and metabolic disorders jointly accelerate this process of vascular aging, making ED an early indicator of systemic vascular disease (12). With advancing age, the body may progressively impair erectile function through cumulative endothelial dysfunction, smooth muscle cell apoptosis, and cavernosal fibrosis, a process that is often irreversible.

The maintenance of erectile function depends on a normal endocrine environment, in which androgens play the most critical role. The hypothalamic-pituitary-gonadal axis regulates the synthesis and secretion of testosterone. With aging, the pulsatile release rhythm of the hypothalamic arcuate nucleus becomes disrupted, the responsiveness of the anterior pituitary to gonadotropin-releasing hormone declines, and the Leydig cells’ response to luteinizing hormone weakens, leading to a progressive decline in serum testosterone levels in men (17, 18). Testosterone deficiency impairs erectile function through multiple mechanisms. It not only drives libido but also modulates erectile function via the NO/cyclic guanosine monophosphate (cGMP) pathway (19). Studies have confirmed that castration or treatment with antiandrogens (e.g., flutamide) can significantly inhibit the activity of constitutive nitric oxide synthase in penile tissue (20). Consequently, in clinical practice, testosterone supplementation is often used as an adjunctive therapy for mild ED; in patients with moderate-to-severe ED, the combined use of testosterone and phosphodiesterase type 5 (PDE5) inhibitors can yield more significant improvement than PDE5 inhibitors alone (3, 21, 22). Several clinical studies have further corroborated the value of testosterone supplementation in ED treatment (23, 24). Notably, this study found a U-shaped nonlinear association between testosterone and vasculogenic ED risk: both low and excessively high levels were associated with elevated risk, whereas moderate levels showed a relative protective trend. This finding is consistent with the threshold-dependent effect of testosterone on the vascular endothelium (25).

Notably, this study preliminarily identified through multiple machine learning approaches that the HAMA score (anxiety) may be associated with the occurrence of vasculogenic ED, a finding that warrants in-depth discussion. Psychological factors play a complex role in ED: they can serve as the core etiology of primary psychogenic ED, or as secondary factors that exacerbate the clinical manifestations of organic ED. From a neurobiological perspective, anxiety states may interfere with the normal processing of sexual stimuli by over-activating the amygdala and weakening prefrontal cortical regulation of the limbic system, while simultaneously inhibiting the activation of the mesolimbic dopaminergic reward pathway, thereby reducing sexual motivation and pleasure (26–28). Furthermore, chronic psychological stress can elevate cortisol levels, which in turn suppress the hypothalamic-pituitary-gonadal axis and lower endogenous testosterone levels (29), indirectly affecting vascular endothelial function. However, given the cross-sectional design of this study, the direction of causality cannot be determined: whether the HAMA score is an independent risk factor for vasculogenic ED, a psychological reaction secondary to ED, or both are linked through common upstream mechanisms such as chronic stress remains unclear. In addition, the association between anxiety scores and the “vasculogenic” diagnostic label may involve conceptual overlap—some patients presenting primarily with anxiety may not have significant vascular pathology but could still exhibit abnormal penile CDDU parameters due to autonomic dysfunction induced by psychological factors. Therefore, when incorporating the HAMA score into the risk factor profile of vasculogenic ED, it should be understood as reflecting a possible complex bidirectional interaction between psychological factors and vascular pathology, rather than a direct causal contribution. Despite this, the finding holds clinical implications: psychological screening should not be overlooked in the assessment of vasculogenic ED; for patients with prominent anxiety symptoms, combined anxiety intervention may still serve as an auxiliary strategy in comprehensive management, even if CDDU indicates vascular pathology.

Furthermore, hypertension (30), smoking (31), and diabetes mellitus (32), as traditional vascular endothelial risk factors, also showed significant positive associations in this study. The inter-individual heterogeneity of their effects was considerable, which may be related to disease duration, degree of control, and concomitant medications. Among them, the SHAP contribution of diabetes mellitus was particularly prominent, suggesting that glycemic control is a key modifiable target in the management of vasculogenic ED.

This study has several important limitations. First, the single-center, retrospective, cross-sectional design, along with the sampling of hospitalized patients, inevitably introduced selection bias, restricting the generalizability of the findings to community or outpatient populations. Although a consecutive enrollment strategy was employed, bias could not be completely eliminated. Second, penile CDDU as the gold standard is operator-dependent, which may affect the accuracy of outcome classification. Third, all machine learning models were trained with default parameters without hyperparameter optimization, potentially resulting in suboptimal model performance and affecting the fairness of comparisons among different models. This study was a preliminary exploratory analysis; in future large-sample multicenter studies, we will adjust the relevant machine learning parameters. Fourth, the model’s predictive performance was only moderate (AUC = 0.682), indicating that it currently lacks the capability for independent clinical decision-making and its value is limited to exploratory research. Fifth, the feature selection process may have omitted some potentially important variables (e.g., medication history, specific disease duration). Sixth, although SHAP analysis revealed nonlinear associations, the cross-sectional design dictates that all interpretations must be confined to associations rather than causality. Finally, the clinical translation of certain nonlinear relationships requires cautious interpretation in combination with domain-specific knowledge.

## 5 Conclusions

This study screened seven key factors associated with vasculogenic ED based on routine clinical indicators, including age, hypertension, smoking, diabetes mellitus, Hamilton Anxiety Scale (HAMA) score, total testosterone, and estradiol. The constructed Random Forest model preliminarily demonstrated potential for identifying individuals at high risk of vasculogenic ED; however, its discriminative ability was moderate and currently insufficient to support independent clinical decision-making. These findings provide exploratory data support for optimizing the clinical evaluation pathway and rationally selecting candidates for CDDU. Future multicenter, prospective studies with more rigorous designs, along with hyperparameter optimization and external validation of the model, are warranted to further evaluate its generalizability and clinical utility.

## Data Availability

The original contributions presented in the study are included in the article/supplementary material. Further inquiries can be directed to the corresponding authors.

## References

[1] Pang K Pan D Xu H Ma Y Wang J Xu P . Advances in physical diagnosis and treatment of male erectile dysfunction. Front Physiol. (2023) 13:1096741. doi: 10.3389/fphys.2022.1096741. PMID: 36699684 PMC9868413

[2] NIH Consensus Development Panel on Impotence . Impotence. JAMA. (1993) 270:83–90. 8510302

[3] Salonia A Bettocchi C Boeri L Capogrosso P Carvalho J Cilesiz NC . European association of urology guidelines on sexual and reproductive health-2021 update: male sexual dysfunction. Eur Urol. (2021) 80:333–57. doi: 10.1016/j.eururo.2021.06.007. PMID: 34183196

[4] Belew D Klaassen Z Lewis RW . Intracavernosal injection for the diagnosis, evaluation, and treatment of erectile dysfunction: a review. Sex Med Rev. (2015) 3:11–23. doi: 10.1002/smrj.35. PMID: 27784568

[5] Feuerriegel S Frauen D Melnychuk V Schweisthal J Hess K Curth A . Causal machine learning for predicting treatment outcomes. Nat Med. (2024) 30:958–68. doi: 10.1038/s41591-024-02902-1. PMID: 38641741

[6] Kokori E Patel R Olatunji G Ukoaka BM Abraham IC Ajekiigbe VO . Machine learning in predicting heart failure survival: a review of current models and future prospects. Heart Fail Rev. (2025) 30:431–42. doi: 10.1007/s10741-024-10474-y. PMID: 39656330

[7] Russo M Nardini D Melchiorre S Ciprietti C Polito G Punzi M . Predicting conversion in cognitively normal and mild cognitive impairment individuals with machine learning: is the CSF status still relevant? Alzheimers Dement. (2025) 21:e14398. doi: 10.1016/j.jns.2025.123929. PMID: 39887916 PMC11848327

[8] Huang W Huang S Fang Y Zhu T Chu F Liu Q . AI-powered mining of highly customized and superior ESIPT-based fluorescent probes. Adv Sci. (2024) 11:e2405596. doi: 10.1002/advs.202405596. PMID: 39021325 PMC11425259

[9] Chen J Ren J Yan P . Application and value of color Doppler ultrasonography in the diagnosis of vasculogenic erectile dysfunction. J China Clinic Med Imaging. (2007) 18:808–10. doi: 10.3969/j.issn.1008-1062.2007.11.014

[10] Canguven O Al-Malki AH Majzoub A . Serum testosterone status in men with penile corporoveno-occlusive dysfunction. Aging Male. (2020) 23:1227–31. doi: 10.1080/13685538.2020.1742682. PMID: 32281465

[11] Kwiendacz H Huang B Chen Y Janota O Irlik K Liu Y . Predicting major adverse cardiac events in diabetes and chronic kidney disease: a machine learning study from the Silesia Diabetes-Heart Project. Cardiovasc Diabetol. (2025) 24:76. doi: 10.1186/s12933-025-02615-w. PMID: 39955553 PMC11829423

[12] Mola JR . Erectile dysfunction in the older adult male. Urol Nurs. (2015) 35:87–93. doi: 10.7257/1053-816x.2015.35.2.87 26197627

[13] Zhong K Hu H Xiao L Fan G Zhang L . Vascular aging-driven erectile dysfunction: pathophysiological mechanisms and emerging therapies-a narrative review. Transl Androl Urol. (2025) 14:4033–47. doi: 10.21037/tau-2025-581 PMC12779443 41522304

[14] Seals DR Jablonski KL Donato AJ . Aging and vascular endothelial function in humans. Clin Sci (Lond). (2011) 120:357–75. doi: 10.1042/cs20100476. PMID: 21244363 PMC3482987

[15] Oliveira AC Cunha PMGM Vitorino PVO . Vascular aging and arterial stiffness. Arq Bras Cardiol. (2022) 119:604–15. doi: 10.5935/abc.20170091. PMID: 36287415 PMC9563886

[16] Wespes E . Smooth muscle pathology and erectile dysfunction. Int J Impot Res. (2002) 14:S17–21. doi: 10.1038/sj.ijir.3900792. PMID: 11850730

[17] De Silva NL Papanikolaou N Grossmann M Antonio L Quinton R Anawalt BD . Male hypogonadism: pathogenesis, diagnosis, and management. Lancet Diabetes Endocrinol. (2024) 12:761–74. doi: 10.1016/s2213-8587(24)00199-2. PMID: 39159641

[18] Pitteloud N Hardin M Dwyer AA Valassi E Yialamas M Elahi D . Increasing insulin resistance is associated with a decrease in Leydig cell testosterone secretion in men. J Clin Endocrinol Metab. (2005) 90:2636–41. doi: 10.1210/jc.2004-2190. PMID: 15713702

[19] Besong EE Ashonibare PJ Akhigbe TM Obimma JN Akhigbe RE . Sodium acetate abates lead-induced sexual dysfunction by upregulating testosterone-dependent eNOS/NO/cGMP signaling and activating Nrf2/HO-1 in male Wistar rat. Naunyn Schmiedebergs Arch Pharmacol. (2024) 397:1233–43. doi: 10.1007/s00210-023-02696-y. PMID: 37658211

[20] Penson DF Ng C Cai L Rajfer J González-Cadavid NF . Androgen and pituitary control of penile nitric oxide synthase and erectile function in the rat. Biol Reprod. (1996) 55:567–74. doi: 10.1095/biolreprod55.3.567. PMID: 8862773

[21] Bhasin S . Testosterone replacement in aging men: an evidence-based patient-centric perspective. J Clin Invest. (2021) 131:e146607. doi: 10.1172/jci146607. PMID: 33586676 PMC7880314

[22] Gul M Serefoglu EC . An update on the drug safety of treating erectile dysfunction. Expert Opin Drug Saf. (2019) 18:965–75. doi: 10.1080/14740338.2019.1659244. PMID: 31433252

[23] Snyder PJ Bhasin S Cunningham GR Matsumoto AM Stephens-Shields AJ Cauley JA . Effects of testosterone treatment in older men. N Engl J Med. (2016) 374:611–24. doi: 10.1210/endo-meetings.2011.part3.p27.p3-209. PMID: 26886521 PMC5209754

[24] Rosen RC Wu F Behre HM Porst H Meuleman EJH Maggi M . Quality of life and sexual function benefits of long-term testosterone treatment: longitudinal results from the Registry of Hypogonadism in Men (RHYME). J Sex Med. (2017) 14:1104–15. doi: 10.1016/j.jsxm.2017.07.004. PMID: 28781213

[25] Kataoka T Fukamoto A Hotta Y Sanagawa A Maeda Y Furukawa-Hibi Y . Effect of high testosterone levels on endothelial function in aorta and erectile function in rats. Sex Med. (2022) 10:100550. doi: 10.1016/j.esxm.2022.100550. PMID: 35939869 PMC9537240

[26] Melis MR Succu S Mascia MS Cortis L Argiolas A . Extra-cellular dopamine increases in the paraventricular nucleus of male rats during sexual activity. Eur J Neurosci. (2003) 17:1266–72. doi: 10.1046/j.1460-9568.2003.02558.x. PMID: 12670314

[27] Melis MR Succu S Sanna F Boi A Argiolas A . Oxytocin injected into the ventral subiculum or the posteromedial cortical nucleus of the amygdala induces penile erection and increases extracellular dopamine levels in the nucleus accumbens of male rats. Eur J Neurosci. (2009) 30:1349–57. doi: 10.1111/j.1460-9568.2009.06912.x. PMID: 19769589

[28] Bratzu J Bharatiya R Manca E Cocco C Argiolas A Melis MR . Oxytocin induces penile erection and yawning when injected into the bed nucleus of the stria terminalis: a microdialysis and immunohistochemical study. Behav Brain Res. (2019) 375:112147. doi: 10.1016/j.bbr.2019.112147. PMID: 31408664

[29] Kupelian V Shabsigh R Travison TG Page ST Araujo AB McKinlay JB . Is there a relationship between sex hormones and erectile dysfunction? Results from the Massachusetts Male Aging Study. J Urol. (2006) 176:2584–8. doi: 10.1016/j.juro.2006.08.020. PMID: 17085164

[30] Durante A Mazzapicchi A Baiardo Redaelli M . Systemic and cardiac microvascular dysfunction in hypertension. Int J Mol Sci. (2024) 25:13294. doi: 10.3390/ijms252413294. PMID: 39769057 PMC11677602

[31] Ishida M Sakai C Kobayashi Y Ishida T . Cigarette smoking and atherosclerotic cardiovascular disease. J Atheroscler Thromb. (2024) 31:189–200. doi: 10.5551/jat.rv22015. PMID: 38220184 PMC10918046

[32] Goligorsky MS . Vascular endothelium in diabetes. Am J Physiol Renal Physiol. (2017) 312:F266–75. doi: 10.1152/ajprenal.00473.2016. PMID: 27852610 PMC5336585

